# High-resolution record reveals climate-driven environmental and sedimentary changes in an active rift

**DOI:** 10.1038/s41598-019-40022-w

**Published:** 2019-02-28

**Authors:** Lisa C. McNeill, Donna J. Shillington, Gareth D. O. Carter, Jeremy D. Everest, Robert L. Gawthorpe, Clint Miller, Marcie P. Phillips, Richard E. Ll. Collier, Aleksandra Cvetkoska, Gino De Gelder, Paula Diz, Mai-Linh Doan, Mary Ford, Maria Geraga, Jack Gillespie, Romain Hemelsdaël, Emilio Herrero-Bervera, Mohammad Ismaiel, Liliane Janikian, Katerina Kouli, Erwan Le Ber, Shunli Li, Marco Maffione, Carol Mahoney, Malka L. Machlus, Georgios Michas, Casey W. Nixon, Sabire Asli Oflaz, Abah P. Omale, Kostas Panagiotopoulos, Sofia Pechlivanidou, Simone Sauer, Joana Seguin, Spyros Sergiou, Natalia V. Zakharova, Sophie Green

**Affiliations:** 1 School of Ocean and Earth Science, University of Southampton, Southampton, S014 3ZH United Kingdom; 2 Lamont-Doherty Earth Observatory of Columbia University, 61 Route 9W, Palisades, NY 10964 USA; 3 British Geological Survey, The Lyell Centre, Edinburgh, United Kingdom; 4 Department of Earth Science, University of Bergen, Bergen, Norway; 5 Department of Earth, Environmental and Planetary Sciences, Rice University, Houston, USA; 6 Institute for Geophysics, University of Texas at Austin, Austin, USA; 7 School of Earth and Environment, The University of Leeds, Leeds, United Kingdom; 8 Department of Animal Ecology and Systematics, Justus Liebig University, Giessen, Germany; 9 Institut de Physique du Globe de Paris, Sorbonne Paris Cité, Université Paris Diderot, Paris, France; 10 Departamento Geociencias Marinas y Ordenación del Territorio, Facultad de Ciencias del Mar, Universidad de Vigo, Vigo, Spain; 11 Université Grenoble Alpes, Université Savoie Mont Blanc, CNRS, IRD, IFSTTAR, and ISTerre, Le Bourget-du-Lac, France; 12 CRPG, UMR 7358, France. Also at: Université de Lorraine, ENSG, INP, Nancy, France; 13 Department of Geology, University of Patras, Patras, Greece; 14 Center for Tectonics, Resources, and Exploration (TRaX), Department of Earth Sciences, School of Physical Sciences, University of Adelaide, Adelaide, Australia; 15 Géosciences Montpellier, Université de Montpellier, Montpellier, France; 16 University of Hawai’i at Manoa, Hawai’i Institute of Geophysics and Planetology, Honolulu, USA; 17 University Centre for Earth and Space Sciences, University of Hyderabad, Hyderabad, India; 18 Universidade Federal de São Paulo, Departamento de Ciências do Mar, Sao Paulo, Brazil; 19 Department of Geology and Geoenvironment, National and Kapodistrian University of Athens, Athens, Greece; 20 School of Geography, Geology and the Environment, University of Leicester, Leicester, United Kingdom; 21 School of Energy Resources, China University of Geosciences, Beijing, China; 22 School of Geography Earth and Environmental Sciences, University of Birmingham, Birmingham, United Kingdom; 23 Department of Physical Sciences, Kingsborough Community College, City University of New York, New York, USA; 24 Laboratory of Geophysics and Seismology, Technological Educational Institute of Crete, Irakleio, Greece; 25 Graduate School “Human Development in Landscapes”, Christian-Albrechts-Universität zu Kiel, Kiel, Germany; 26 Department of Geology and Geophysics, Louisiana State University, Baton Rouge, USA; 27 Institute of Geology and Minerology, University of Cologne, Cologne, Germany; 28 Ifremer, Department of Marine Geosciences, Centre Bretagne, Plouzané, France; 29 Institute for Ecosystem Research, Christian-Albrechts-Universitat zu Kiel, Kiel, Germany; 30 Department of Earth and Atmospheric Sciences, Central Michigan University, Mount Pleasant, USA

**Keywords:** Geochemistry, Geomorphology, Palaeontology, Sedimentology, Tectonics

## Abstract

Young rifts are shaped by combined tectonic and surface processes and climate, yet few records exist to evaluate the interplay of these processes over an extended period of early rift-basin development. Here, we present the longest and highest resolution record of sediment flux and paleoenvironmental changes when a young rift connects to the global oceans. New results from International Ocean Discovery Program (IODP) Expedition 381 in the Corinth Rift show 10s–100s of kyr cyclic variations in basin paleoenvironment as eustatic sea level fluctuated with respect to sills bounding this semi-isolated basin, and reveal substantial corresponding changes in the volume and character of sediment delivered into the rift. During interglacials, when the basin was marine, sedimentation rates were lower (excepting the Holocene), and bioturbation and organic carbon concentration higher. During glacials, the basin was isolated from the ocean, and sedimentation rates were higher (~2–7 times those in interglacials). We infer that reduced vegetation cover during glacials drove higher sediment flux from the rift flanks. These orbital-timescale changes in rate and type of basin infill will likely influence early rift sedimentary and faulting processes, potentially including syn-rift stratigraphy, sediment burial rates, and organic carbon flux and preservation on deep continental margins worldwide.

## Introduction

Active continental rift zones generate rapidly subsiding basins with significant accumulations of sediments. These settings are thought to be highly sensitive to the interplay of extensional tectonics, sedimentary processes, climate and sea level change^1,2^. Rift basins and other basins in active environments close to sea level are known to experience dramatic changes in environment due to glacio-eustatic fluctuations^3,4^. Both pronounced environmental change and active faulting are thought to control sediment delivery and accumulation in active basins^1–3^. This includes changes in temperature, precipitation and amount and type of vegetation cover affecting erosion and sediment flux, and tectonics driving changes in catchment relief and area, erosion of different lithologies and shifting loci of sedimentation within a basin. However, the paucity of high-resolution constraints makes it difficult to isolate each of these contributions. Erosion and deposition of sediments also appear to have profound effects on rift localisation, fault longevity, crustal creation, and thermal structure^5–9^. However, the record of these processes is deeply buried and inaccessible in most ancient and mature rifts; high resolution data on age and rates of process are not available for deep and old sediments, the spatial resolution of seismic data is low, and sections are deeply buried and often overprinted by later deformational phases so do not preserve information on early fault history and its link to sedimentary processes. Basins in active environments tend to only have very short (mostly <15–25 ka) records from piston cores. Rare longer temporal records exist (Black Sea, Lake Malawi^2,10^) but in environments of lower tectonic activity or without ocean connection. Therefore, our ability to constrain in sufficient resolution temporal and spatial variations in basin paleoenvironment, sedimentation patterns or rift development, or the role of climate and sea level in moderating these factors is severely restricted. As a result, many hypotheses tend to be model derived and remain untested.

**Figure 1:**
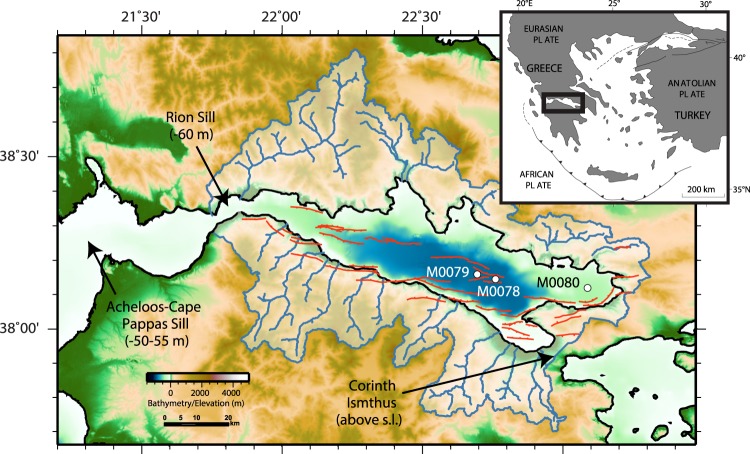
Map of the Gulf of Corinth and Corinth Rift system. Includes primary active rift faults (after ref.^17^), positions of bounding sills to the Gulf, IODP drillsites, and primary catchment areas and rivers feeding the offshore basin (from ref.^42^). Inset shows regional setting.

The Corinth Rift (Fig. 1) is a region of rapid, localised extension and high seismic activity. Current extension rates reach 10–15 mm/yr^11–13^, some of the highest in the world. Corinth’s high rates of tectonic activity, high sediment fluxes, closed drainage system and preservation of the syn-rift record make it a unique laboratory for the study of extension, sedimentation and paleoenvironment in a young rift.

Rifting began ~5 Ma with 3 main phases identified by integration of onshore depositional records and offshore seismic stratigraphy^1,14–21^. Following an early continental and lacustrine phase (phase 1), depocentre deepening occurred ~2 Ma (phase 2) resulting in increased subsidence and sediment supply at the location of the modern Gulf of Corinth. At ~0.5–0.8 Ma, fault activity generally stepped northward, establishing the dominant N-dipping rift fault system along the southern boundary (Fig. 1) and development of fan deltas along the western part of the southern Gulf margin (phase 3). The transition from Phase 2 to 3 may mark the onset of repeated connection to the open ocean, although earlier marine incursions are recognised onshore^21^.

From onshore deposits and seismic imaging of offshore stratigraphy, the rift basin environment during phase 3 has been interpreted to alternate between marine conditions during interglacial/highstand periods and isolated from the open ocean during glacial/lowstand periods, as eustatic sea level fluctuated relative to the boundaries of the basin in the Late Quaternary^17–19,21–25^. Sills at the western mouth of the Corinth Basin (Rion sill: ~60 m; Acheloos-Cape Pappas sill: ~50–55 m) currently control the connection with the Ionian Sea (Fig. 1) and are interpreted to control connection for at least the last 200 kyr^22,26,27^. Prior to this, the cyclical connection to the ocean was either controlled by these sills in the west or by one or more sills in the east, primarily the Corinth Isthmus (Fig. 1), currently above sea level but submerged in the past^28^. Prior to IODP Expedition 381, the only data available to constrain paleoenvironment evolution, and thus to examine alternating conditions, were shallow piston cores recovering sediments no deeper than 30 metres below seafloor (mbsf) and no older than 50 ka^29,30^. These data showed a change in sediment facies and micropaleontological assemblages at ~12 ka, marking the timing of transgression above the basin sill following the last glacial maximum. To date, these short records and similar records from other active basins do not provide a sufficiently long-duration record to evaluate sea-level driven changes in environment and their implications for sedimentation and rift processes.

IODP Expedition 381 drilled and cored the most recent ~1–2 Myr of syn-rift sediments to a depth of 705 mbsf, in October-December, 2017 (Fig. 1)^31^. This is the longest and highest resolution record of its kind in a young extensional basin at the point of connection to the global oceans, and it provides the first constraints on the age of the full rift sequence, syn-rift stratigraphy, rates and timings of rift tectonic processes, sediment fluxes and basin environmental conditions. Here we use this new record to test the hypothesis that glacial-interglacial timescale (10’s-100 kyr) climatic and environmental change strongly influences the nature and volume of accumulating sediment within the Corinth basin. If such change occurs, we explore how sediment delivery and accumulation are affected, including effects on sediment fluxes, grain size and lithostratigraphy. Alternatively, the basin is insensitive to regional and local short-term environmental and climatic change being, instead, dominated by longer term tectonic-driven changes. Finally, we hypothesise that regional climatic change at 10–100 kyr timescales results in major basin environmental change with a corresponding change in aquatic biota.

## Results

**Figure 2:**
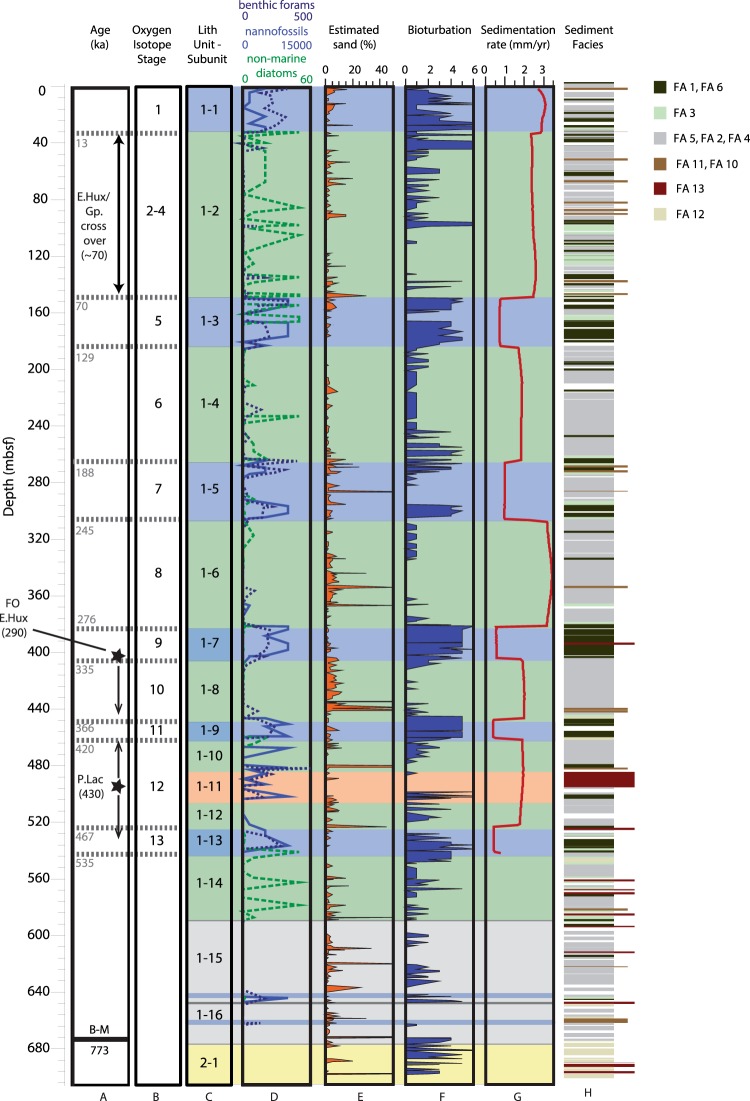
Results from Site M0079. (**A**) Age constraints from calcareous nannofossils and magnetostratigraphy (black text) and interpreted ages from correlation of basin environment with eustatic sea level curve (grey dotted lines and text, see Methods). (**B**–**M**) Brunhes-Matuyama chron boundary; E. Hux.: first occurrence (FO) of *E. huxleyi*; P.Lac: last occurrence (LO) of *P. lacunosa;* E.Hux/Gp. crossover: crossover in dominance between *E. huxleyi* and *Gephyrocapsa*. Arrows indicate potential uncertainty of true depth position of age marker (see text and Methods), and age is given in parentheses; (**B**) Oxygen isotope stages (OIS); (**C**) Lithostratigraphic units and subunits, coloured by basin environment interpretation (blue: marine; green: isolated/semi-isolated; grey: undetermined), subunit 1–11 is slumped interval (orange), yellow denotes Unit 2; (**D**) Abundance/counts of calcareous nannofossils (light blue), benthic foraminifer (dark blue, dotted) and non-marine diatoms (green, dashed); (**E**) Estimated sand percent; (**F**) Bioturbation intensity; (**G**) Decompacted sedimentation rate (see Methods); and H) Sediment lithology coloured according to facies association, longer bars representing coarser intervals (Table S3; ref.^31^).

Three sites were drilled during Expedition 381 (Fig. 1). Site M0079, the focus of observations in this paper, is in the central Corinth basin, the primary depocentre of the Gulf of Corinth (Fig. S4). The nature of the site, within a primary locus of sedimentation, is based on analysis of syn-rift sediment thicknesses from integration of seismic profiles around the rift^17^. This site samples an expanded section of the most recent rift phase (phase 3), and thus provides a high-resolution record of extension, sedimentation and sea level change over the last ~750 ka. The site contains a thick, continuous succession of fine-grained distal facies and no faults. Thus, the depositional history including sediment accumulation rates (both absolute and variations through time) from this borehole are representative of the Corinth primary depocentre. The upper lithostratigraphic Unit 1, the focus of this paper, is subdivided into 16 subunits and has its base at 677 mbsf (Fig. 2), with a thin Unit 2 section beneath.

### Evidence for cyclical rift basin paleoenvironment

**Figure 3:**
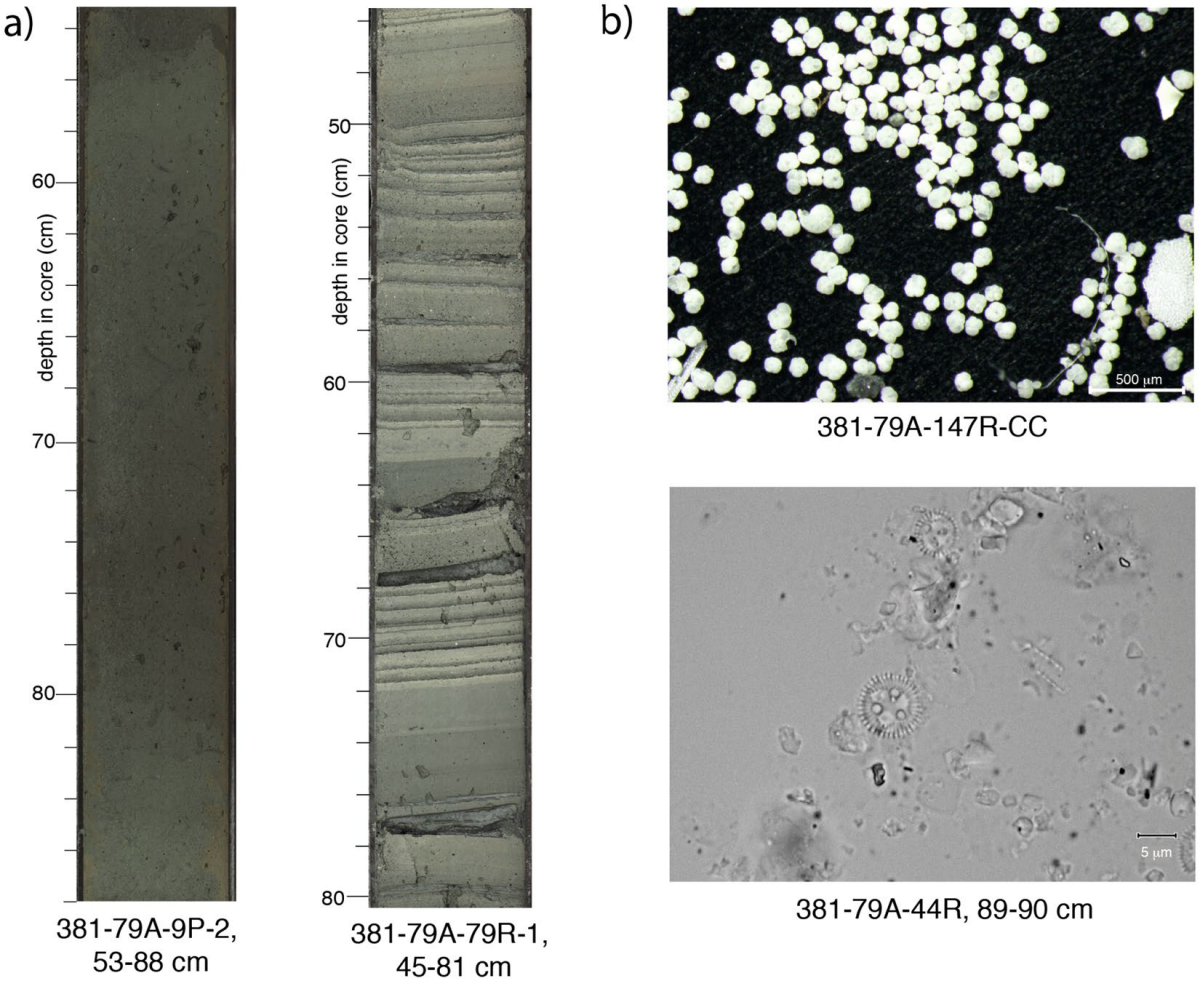
Core images. (**a**) Core section examples (from Site M0079) of typical sedimentary facies in marine (left; Facies Association 1, homogeneous mud) and isolated (right; Facies Association 4, laminated greenish grey to grey mud with mud beds) intervals; (**b**) Typical microfossil assemblages from marine (above) and isolated (below) intervals (from Site M0079). Marine assemblage image shows planktic forams from >125 μm fraction; isolated assemblage (at 1000x magnification) shows non-marine planktonic diatom taxon *Pantocsekiella ocellata*.

Microfossil assemblages in Unit 1 alternate primarily between “marine” assemblages composed exclusively of marine microfossils, and assemblages with significant amounts of both non-marine and marine microfossils that reflect a range of complex conditions that are neither fully marine nor freshwater, with different degrees of marine influence (hereafter, “isolated”). During marine intervals, both marine and terrestrial microfossils are abundant, including calcareous nannofossils, marine diatoms, planktic and benthic foraminifera, dinoflagellate cysts, foraminifera test linings, pollen and spores (Figs 2 and 3, Table S1). During isolated intervals, non-marine diatoms are typically present in moderate to high abundances (Figs 2 and 3, Table S1), and observed in combination with moderate to high abundances of green algae coenobia and spores, marine or brackish dinoflagellate cysts, pollen and spores and low abundances of marine microfossils. The variability of microfossil and palynomorph assemblages within and between marine and isolated intervals directly indicates changes in aquatic basin paleoenvironment (e.g., salinity) and in terrestrial environment around the rift basin, paleoclimate and depositional setting. Based on these assemblages, the 16 subunits of Unit 1 were identified as marine or isolated intervals as a function of connection to or isolation from the open ocean. A combination of lithology, microfossil assemblages and core physical properties data provided the basis for pinpointing the subunit boundaries^31^.

### Mid-Late Quaternary Chronostratigraphy

Biostratigraphy and magnetostratigraphy provide age control that allows us to link changing paleoenvironment to the eustatic sea level curve (Fig. 2). Calcareous nannofossils in the marine intervals confirm that Unit 1 is Middle Pleistocene through Recent based on (1) the Last Occurrence (LO) of *Pseudoemiliania lacunosa* (0.43 Ma) at 496 mbsf; (2) the First Occurrence (FO) of *Emiliania huxleyi* (0.29 Ma) at 405 mbsf; and (3) the crossover in dominance between *E. huxleyi* and *Gephyrocapsa* “small” (<3 µm) within subunit 1–2 (Fig. 2), documented at ~70 ka in the Mediterranean^32^. Due to the fluctuating environment within the basin, the syn-rift stratigraphy does not contain a continuous marine section, thus the LO of *P. lacunosa* and FO of *E. huxleyi* may not mark the true respective LO and FO (see Methods). However, the observed specimens are well preserved and moderately to highly abundant, and support other age markers. Magnetostratigraphy indicates the Brunhes-Matuyama chron boundary (0.773 Ma) occurs at ~665 mbsf at the base of Unit 1 (Fig. 2).

The overall pattern of downhole cyclicity of the Unit 1 subunits (marine vs isolated) strongly supports the paleoenvironment being predominantly controlled by eustatic sea level fluctuation; tectonic control of this cyclical nature can be ruled out. In addition, the marine phases include short-lived isolated intervals, and vice versa, as expected from details of the eustatic fluctuations (e.g., within subunit 1–2, Fig. 2). We correlated the transitions between marine and isolated intervals to the eustatic sea level curve of Spratt and Lisecki^33^ assuming a bounding sill depth of −60 m (present day Rion sill depth^27^) and that flooding of this sill marks the transition between isolated and marine conditions (Fig. 2). This correlation and a sill depth of −60 m agree with age constraints from bio- and magnetostratigraphy and work exceptionally well to ~545 mbsf and the base of subunit 1–13, which is correlated with oxygen isotope stage (OIS) 13 at ~535 ka (Fig. 2). Below this depth, marine fauna occur in thin stratigraphic intervals within subunits 1–15 and 1–16 (Fig. 2). This may be explained by the basin-controlling sill being at a shallower level at this earlier stage, resulting in a limited record of earlier highstands in the basin.

Currently the primary age uncertainty derives from the presumption of a constant sill depth. As above, the nature of the marine record in the deeper part of Unit 1 suggests the sill was potentially shallower at this time. However, the persistence of the marine intervals and their relatively consistent thickness until we reach the deeper part of Unit 1 gives strong support to the controlling sill remaining close to this depth, supported by previous studies^27^. A significant shift of the sill to shallower depths (e.g., 0–20 m) would severely restrict marine incursions, and this is not observed (unless we invoke very significant differences in sedimentation rate between marine intervals with time). Shallowing the sill level by up to 30 m does not significantly change the inferred age model, and the relative differences in sedimentation rates between isolated and marine intervals persist (see Methods, Fig. S3). In the absence of additional constraints, a constant sill depth is the most conservative assumption. As further research is undertaken on these drill cores, we will be able to add further absolute age constraints, including from tephra that are starting to be identified. This will reduce uncertainties of the age model and in turn provide constraints on sill height.

### Syn-rift sedimentary stratigraphy and processes

Fine-grained (mud dominated), carbonate-rich turbiditic and hemipelagic sediments dominate the syn-rift succession of the Corinth Rift basin. However, we observe significant differences in lithology and depositional processes between the marine and isolated intervals of lithostratigraphic Unit 1. Marine subunits of Site M0079 are moderately to highly bioturbated and dominated by homogeneous to poorly-bedded greenish grey muds (Figs 2 and 3a). Biogenic material is common and comprises calcareous nannofossils, foraminifera and marine diatoms, and fragments of gastropods and bivalves. Marine subunits are also characterised by increased total organic carbon (TOC) concentration with respect to the isolated subunits^34^. Isolated subunits are characterised by laminated to thinly bedded and homogeneous grey and greenish grey muds (Fig. 3a), some with black, organic-rich laminations and beds, and are generally lacking or having sparse bioturbation (Fig. 2). Overall, isolated intervals contain a higher proportion of relatively coarser grained lithologies (silts and sands). This is particularly well expressed in the deeper subunits of Unit 1, for example compare sand content of marine subunits 1–5, 1–7 and 1–9 with isolated subunits 1–6 and 1–8 (Fig. 2).

We calculated sedimentation rates for each marine and isolated subunit using decompacted thicknesses of total sediment (hemipelagic plus gravity flow deposit) derived using borehole porosity data (see Methods)^31^. The primary uncertainties in these calculations include the sill depth, which controls the precise estimated timing of the transition between marine and isolated intervals and their duration, potential differences in seafloor porosity in the past, and the applicability of the decompaction curve used, particularly for the marine versus isolated intervals, which may compact differently (see Methods). Unit 1 has an average sedimentation rate of 1.1 mm/yr, but a striking result is the clear difference in sedimentation rate between the marine and isolated intervals/subunits within Unit 1 (Fig. 2). Sedimentation rates in isolated intervals/subunits (range of averages per interval: 1.7–3.3 mm/yr) are generally higher than those of the marine intervals/subunits (range of averages per interval: 0.3–0.7 mm/yr). For each pair of successive marine-isolated intervals (e.g., between subunits 1–3 and 1–2), excluding the Holocene subunit 1–1, the rates are 1.9–6.7 times greater in the isolated than the marine intervals. This result is supported by indicators^35^ of enhanced fluvial input into the basin during glacial periods. Sedimentation rates for the 0–12 ka (Holocene) marine interval (subunit 1-1) are unusually high (average 2.9 mm/yr) compared to older marine intervals and are more similar to those of the isolated intervals. This suggests that the Holocene section recovered by piston cores in the Gulf of Corinth cannot be used to generalise conditions farther back in time. Overall there is also a slight pattern of increased rates from the past to present for both isolated and marine intervals. Deviating from this pattern, the sedimentation rate is particularly high in subunit 1–6 (MIS 8) at 3.3 mm/yr, and this is considerably higher than the prior and subsequent isolated subunits (Fig. 2).

Translating results from Site M0079 to the thickest Unit 1 section of the whole rift basin (1.5 km versus 0.68 km at Site M0079) using seismic stratigraphic unit thickness^17^ generates a maximum average sedimentation rate for Unit 1 in the basin of ~2 mm/yr and rates up to 3–4 mm/yr over individual ~100 kyr time periods. Overall these results indicate high sediment accumulation rates overall and large differences in sediment accumulation between marine/interglacial and isolated/glacial intervals.

## Discussion

### Changing environment in the Late Quaternary rift basin

The changing environmental conditions reflected by the microfossil assemblages are interpreted to arise from fluctuating eustatic sea level with respect to the bounding sills of the Gulf of Corinth. Many previous studies have hypothesised that alternating marine and isolated conditions in the Gulf are driven by sea level change^15,18,25^, but previous observations from this and other semi-isolated basins (e.g., Sea of Marmara, Black Sea) primarily came from piston cores and thus only sampled part of the most recent glacial cycle and the Holocene^3,4,29,30^. The cores recovered during IODP Expedition 381 provide the first direct evidence of sea-level driven changes in paleoenvironment in an active rift basin over hundreds of thousands of years (0–750 ka).

Observations from Site M0079 demonstrate profound climate and sea-level driven changes in the basin paleoenvironment, sediment accumulation and geochemistry^31,34^ of this young rift over the last ~750 kyr. The microfossil assemblages are highly complex and variable – this is particularly true within the isolated intervals and within the marine-isolated transitions. We also note variations between the successive marine and isolated intervals. This variability suggests a much wider range of basin environments than simple marine versus freshwater end members for the marine and isolated intervals, respectively. The primary controls on the distribution of microfossil assemblages present are salinity, nutrient availability, pH, light, turbidity, temperature, and transport. Salinity, apparently the primary driver, is likely to be controlled by the combined effects of connection to the open ocean, freshwater influx/dilution, precipitation, evaporation, sill overspill from the Mediterranean Sea, and water body stratification and overturning that together result in a variety of conditions as eustatic sea level and climate fluctuated. Further research will investigate the specific environmental conditions indicated by the full suite of microfossils and how these vary through time.

Distinct differences in sedimentary processes and sedimentation rate are also observed between the marine and isolated intervals. The marine intervals are characterised by more homogeneous mud sequences, reduced coarse-grained sediment, and increased TOC concentration^34^. The changes in accumulation of organic carbon between marine and isolated intervals result from the interplay between basin productivity, terrestrial carbon input, preservation and sediment flux, all driven by cycling climate and basin environment^34^. Finally, the correspondence of increased bioturbation and homogeneity of sediments in the marine intervals indicates increased benthic organism activity. In summary, the clear increase in sedimentation rate during glacial periods when sea level is low and the basin is isolated (Fig. 2) implies that sediment fluxes (rates, volumes and grain size) into the basin from the subaerial rift flanks are increased during glacial times, probably leading to reduced benthic faunal activity within the basin.

### Influences on sediment flux into the basin

Compared to other basins at similar evolutionary stages and over comparable time periods, sedimentation rates in Corinth (up to ~3–4 mm/yr) are high (e.g., Sea of Marmara, Lake Baikal, Malawi, Northern Gulf of California^2,3,36,37^). The Northern Gulf of California and Sea of Marmara have comparable sedimentation rates (up to 2–4 mm/yr^3,37^). In Corinth, sediment flux is primarily controlled by combinations of high uplift rates of the rift flanks (Late Quaternary rates of 1–5 mm/yr^15,38–41^) and erosion of weakly consolidated/lithified materials into accommodation space created through rapid extension and subsidence. The ability of the fluvial systems to transport sediment is also an important control on delivery of sediments to the basin. However, in the Corinth system, fluvial systems do not appear to be limited by their ability to transport sediment^38^, and thus the increased sediment fluxes during glacial periods reflect increased sediment production and supply.

Many of the most significant catchments are located on the southern rift margin, i.e., the N. Peloponnese (with the exception of the Mornos on the NW rift margin), coincident with highest uplift rates in the footwall of the southern rift boundary N-dipping fault system^1^ (Fig. 1). In spite of the exceptionally high sedimentation rates shown here, the Corinth basin is presently underfilled, and it is likely that it has been underfilled for many 100’s of kyr and potentially at least 1.5 Myr based on the height of delta foresets on the Gulf margins and those exposed onshore^21^. This highlights that rift basin subsidence rates exceed sediment accumulation rates, even during glacial/lowstand periods when sediment flux into the basin is enhanced by a factor of 2–7. This may be amplified by catchment averaged erosion rates being insufficient to keep pace with uplifting topography.

Although tectonic subsidence and uplift clearly control the larger-scale development of the basin, they would not be expected to fluctuate cyclically on timescales of 10’s kyr, as sedimentation rates fluctuate here. Therefore, we propose that basin sediment accumulation, including variations between glacial and interglacial periods, is a function of one or more of the following: (a) climate (temperature, precipitation) controlling erosion rates and sediment flux^29,42^; (b) vegetation type and cover changing runoff and sediment retention/erosion rates; c) changing basin salinity driving more efficient hyperpycnal flows during isolated/glacial intervals^1^. Because the shelf is narrow in much of the Gulf of Corinth, enhanced slope failure due to direct sediment supply to the shelf edge is unlikely to contribute and catchments are unlikely to significantly increase in area, during sea level lowstands. However, the fluvial and deltaic material exposed on the shelf during lowstands may be more easily erodable, and the partially exposed, relatively unstable margin slopes may be more susceptible to failure^27^. A previous study proposed that a combination of cool, wet winters in the last glacial and reduction in tree cover led to increased sediment availability and flux during glacials estimated over the last ~100 kyr in the eastern Corinth rift^29^. However, pollen-based precipitation reconstructions from the region^43–45^ indicate a decrease in precipitation during glacials, not an increase. Long pollen records across the Balkan Peninsula suggest interglacial forested landscapes alternating with open vegetation during glacials^44–46^, and our new drill cores in the Corinth basin show the same results. Our new cores also have lower pollen and higher corroded pollen grain concentrations in isolated intervals, the latter indicating increased reworking attributed to increased soil erosion, often linked to open vegetation^47^. These pollen and sedimentation rate results from the new IODP borehole record and previous studies lead us to conclude that both reduction and change in type of vegetation cover resulted in increased soil erosion and higher sediment flux into the basin during the isolated/glacial intervals. Analysis of bedrock fault scarp weathering shows evidence for enhanced physical weathering during glacial periods in the Mediterranean region^48^, which may add to the observed Corinth sediment flux increase. Contrasts in seasonality and storminess between glacials and interglacials may have also contributed to erosion rates, but this parameter has not yet been resolved from our new sedimentary record, or previous studies in the region. Sedimentation rates from piston cores and drilling have also revealed variations in sedimentation rates over glacial/interglacial cycles in the Sea of Marmara and Black Sea^3,10^.

The Holocene (last marine/interglacial interval) appears to have significantly higher sedimentation rates than earlier marine/interglacial intervals and is comparable to isolated/glacial intervals (Fig. 2). Forest clearance and agriculture are visible in pollen records from Greece since the mid-Holocene (~6 ka)^49^, therefore it is likely that human activity in the study region is responsible for the high sediment fluxes observed in Holocene subunit 1–1^50^.

### Implications for the early history of rifts

The Corinth Rift basin provides a unique window into an active rift basin environment at the point in time where the rift basin connects to the global ocean system due to rifting and resulting subsidence. It therefore is intermediate between terrestrial rift basins that have yet to achieve a connection to the open oceans (e.g., Lake Baikal, the majority of the East African Rift system) and rifts that have fully connected and no longer oscillate between open and isolated basin environment (e.g., Gulf of California, passive rifted margins). The new Corinth boreholes provide an extended record with unprecedented resolution over multiple glacio-eustatic cycles and will add insight to how the environment of other semi-isolated basins will have developed over time, such as the Sea of Marmara and Black Sea. Our new results illustrate the significant impact of regional climate at orbital timescales (10 s–100 s kyr) on sediment accumulation, and how this in turn affects patterns of organic matter accumulation, preservation and burial history (see also ref.^34^). We would expect to see similar cyclical patterns of organic carbon to those observed here in other rift basins regularly connecting to the oceans. Observed sedimentation patterns, controlled by sea level and climate, could have implications for the distribution of potential source and reservoir rocks within the early phase of sediment build-up on rifted margins, in particular where icehouse conditions prevailed. This is because sediment build up impacts the burial history, preservation and thermal evolution of deep rifted margin sediments and organic matter. Cyclical fluctuations in sediment accumulation combined with feedbacks on fault activity will have important implications for how tectonics and climate interact to control the stratigraphy of mature rifts and rifted margins worldwide^51^.

The observation of high average rates of sedimentation that vary substantially over ~10–100 kyr timescales could have important consequences for tectonic processes in Corinth and early rifting in general. Surface processes can impact lithospheric extensional processes, in particular, the redistribution of mass from the uplifted footwalls into the basin promotes greater extension on rift faults before they are abandoned in favour of new faults^9^. Thicker sediments could also reduce differences in buoyancy forces resulting from thinning, and thus promote narrow rifting^5^. Many of these inferences are based on modelling or larger scale structure but with limited temporal constraints. For the relatively high slip rates on faults bounding the Corinth Rift (2–10 mm/yr)^15^, the temporal changes in erosion onshore and deposition offshore observed here could modulate fault evolution. Further application of the new drilling results in the Corinth rift to analysis of the fault activity will allow us to test whether sedimentation changes, even on relatively short timescales, can impact rift faulting.

## Conclusions

A new scientific drill core record from the syrift sequence of the active Corinth rift provides the first direct observations that basin environment fluctuated between marine conditions during eustatic highstands and isolated conditions during eustatic lowstands when the basin is cut off by bounding sills over the last ~700 ky. Sedimentation rates in the basin show significant variations on 10’s-100 kyr timescales and are markedly increased during glacial/isolated periods. In contrast, bioturbation and organic carbon concentrations are reduced during glacial/isolated periods. The new borehole data, supported by other studies, suggest sedimentation rate changes are a function of a decrease and change in type of vegetation cover in glacial periods, resulting in increased erosion and basin sediment flux. The aquatic basin environment is clearly influenced by 10–100 kyr sea level fluctuation and climate, with the microfossil assemblages sampled indicating much greater complexity than simple alternations between fully marine to freshwater conditions. These results thus reveal the dominant role of climate and sealevel change in generating ~100-ky variations in sedimentation rate and basin environment in this active rift basin.

## Methods

### Micropaleontology and Basin Environment

Microfossils (calcareous nannofossils, marine and non-marine diatoms, planktic and benthic foraminifer, dinoflagellate cysts, foraminifer test linings, freshwater algae coenobia and spores, and aquatic pollen and spores) were used to distinguish basin paleoenvironment, principally differentiating “marine” or “isolated”. Supplementary Table S1 gives abundances of key microfossil groups: calcareous nannofossils and benthic foraminifer (marine indicators) and non-marine diatoms (isolated indicators) with depth in Hole M0079A. Qualitative counts of calcareous nannofossils and non-marine diatoms are based on the Cascading Count Method^52^. The numerical approximations associated with abundance in Fig. 2 (abundance: Barren (B), Very Rare (VR), Rare (R), Few (F), Common (C) and Abundant (A)) for calcareous nannofossils and non-marine diatoms are outlined below:

Calcareous Nannofossils: B = 0; VR = 1–5; R = 6–100; F = 101–1500; C = 1501–5000; A = 5001–10,000+

Non-marine Diatoms: B = 0; VR = 1; R = 2; F = 3–10; C = 11–20; A = 21–50+

The abundance of benthic foraminifer is represented by the number of individuals found in ~10 cc of wet sediment. Benthic foraminifer were counted in the >125 µm fraction. In the isolated intervals, where abundance in the >125 µm is generally lower than 10 individuals, the 63–125 µm fraction was also screened.

### Age Model

Age constraints were from shipboard biostratigraphic (calcareous nannofossils) and magnetostratigraphic analyses. Biozonation of calcareous nannofossils was applied using existing studies^53,54^. The website www.mikrotax.org aided identification of calcareous nannofossils. Calcareous nannofossils provide three age markers (shown on Fig. 2). Due to the fluctuating environment within the basin, the syn-rift stratigraphy does not contain a continuous marine section, thus the LO of *P. lacunosa* and FO of *E. huxleyi* may not mark the true respective LO and FO. We note that *P. lacunosa* was identified in a coherent interval within a large-scale slump that defines subunit 1–11 (Fig. 2)^31^ surrounded by intervals interpreted as isolated. The stratigraphic interval containing this marine calcareous nannofossil assemblage is intact with no evidence for reworking. Therefore, this coherent interval of slumped sediments containing *P. lacunosa* is interpreted to represent a part of an older marine interval, most likely the time equivalent of the underlying marine subunit 1–13. Preliminary magnetostratigraphy analysis provides one age marker in Hole M0079A, the Brunhes-Matuyama chron boundary at 0.773 Ma at a depth of 665 mbsf (Fig. 2). A total of 532 discrete sediment cubic samples were collected from working halves at intervals of ~1.5 m throughout the borehole. All samples were demagnetised using alternating field (AF) treatment in 14 progressive field steps from 5 to 40 mT (with 5-mT increments) and from 40 to 100 mT (with 10-mT increments). Remanent magnetisation direction and intensity were measured before and after each demagnetization step using the horizontal pass-through super-conducting cryogenic rock magnetometer (SRM 755–4000, 2G Enterprises) at the University of Bremen. The inclination of the remanence after AF demagnetisation at 40 mT was used to determine the polarity of each sample (i.e., normal or reversed) and build a magnetostratigraphy downhole. Magnetozones identified from the data were correlated to the Geomagnetic Polarity Time Scale – GPTS^54,55^.

In addition to the above age markers, the age model was developed by tying the Unit 1 subunit boundaries between the marine and isolated intervals to eustatic sea level (Supplementary Fig. S1 and Table S2^33^). A sill depth of −60 m (the current depth of the Rion sill at the western end of the Gulf of Corinth^27^) was used to determine the transition timing between marine and isolated. See Supplementary Information for further discussion of sill depth and impact of its depth change on sedimentation rates. All ages between these transitions were extrapolated linearly. Below a depth of ~545 mbsf, marine intervals were thin or absent (Fig. 2). Therefore, the age model developed from microfossil-based sea level correlation could not be applied from here to the base of the hole; however, the Brunhes-Matuyama chron boundary at 665 mbsf provides absolute age constraint near the base of the hole. Ages and depths in Hole M0079A are shown in Supplementary Table S2.

### Facies Associations and Bioturbation

The lithostratigraphy of the syn-rift sediments drilled during Expedition 381 were categorised into facies associations (FA^31^) defined by physical and biogenic features of the sediment, including bedding and lamination style, grain size, colour, body and trace fossils. The facies associations used in this paper are defined in Supplementary Table S3 and a simplified version is depicted in Fig. 2. The degree of bioturbation applied to the cores is a semi-quantitative assessment ranging from 0 (no bioturbation) to 6 (completely bioturbated)^56^.

### Sedimentation Rate

Sedimentation rates were calculated using ages described above and decompacted sediment thicknesses. Decompaction was based on porosities measured using the “moisture and density” technique on discrete 6 cm^3^ samples spaced at ~1.5 m on cores from Hole M0079A. The wet and dry masses of these samples were measured before and after being dried in a convection oven at 60° ± 5 °C for 24 hrs, respectively. The volume of dried sample was measured with a helium-displacement pycnometer. These measurements were then used to calculate the volume and mass of water originally in the samples, and the porosity of the samples (Supplementary Table S4^31^).

Next, we determined a smooth porosity function to use for decompaction. We first removed outliers by fitting a 2^nd^ order polynomial to the measured porosities and discarding values with residuals greater than 1.5, and then fit a 35^th^ order polynomial to the remaining points. A high-order polynomial was required to capture the observed variations in porosity between marine and isolated subunits (Supplementary Fig. S2 and Table S5).

The decompacted thickness of sediments from a given depth interval, *T*_i_^*^, was determined (Supplementary Table S5) assuming that there is not alteration of the grains:

$${T}_{i}^{\ast }=\frac{{T}_{i}(1-{\varphi }_{i})}{(1-{\varphi }_{i}^{\ast })}$$

where *T*_*i*_ and *ϕ*_*i*_ are the compacted thickness and porosity of a given interval, respectively, where the porosity is taken from the smoothed function. *ϕ*_i_^*^ is the initial porosity, and is assumed to be 56%, the porosity at the modern seafloor in Hole M0079A (Supplementary Table S4^31^).

## Supplementary information

Supplementary information

Dataset 1 (Table S1)

Dataset 2 (Table S4)

Dataset 3 (Table S5)

## Data Availability

All data and material pertinent to this study is contained within the manuscript and Supplementary Information, and/or the IODP Expedition 381 Preliminary Report (Shillington *et al*., 2019; ref.^31^). The full Expedition Report and dataset from IODP Expedition 381 will become openly available on March 1, 2019 (McNeill *et al.*, 2019; ref.^34^) via the IODP website (https://www.iodp.org/resources/access-data-and-samples), including access to core materials and logging data.
