# Author Correction: High-resolution record reveals climate-driven environmental and sedimentary changes in an active rift

**DOI:** 10.1038/s41598-019-42749-y

**Published:** 2019-04-19

**Authors:** Lisa C. McNeill, Donna J. Shillington, Gareth D. O. Carter, Jeremy D. Everest, Robert L. Gawthorpe, Clint Miller, Marcie P. Phillips, Richard E. Ll. Collier, Aleksandra Cvetkoska, Gino De Gelder, Paula Diz, Mai-Linh Doan, Mary Ford, Maria Geraga, Jack Gillespie, Romain Hemelsdaël, Emilio Herrero-Bervera, Mohammad Ismaiel, Liliane Janikian, Katerina Kouli, Erwan Le Ber, Shunli Li, Marco Maffione, Carol Mahoney, Malka L. Machlus, Georgios Michas, Casey W. Nixon, Sabire Asli Oflaz, Abah P. Omale, Kostas Panagiotopoulos, Sofia Pechlivanidou, Simone Sauer, Joana Seguin, Spyros Sergiou, Natalia V. Zakharova, Sophie Green

**Affiliations:** 1 School of Ocean and Earth Science, University of Southampton, Southampton, S014 3ZH United Kingdom; 2 Lamont-Doherty Earth Observatory of Columbia University, 61 Route 9W, Palisades, NY 10964 USA; 3 British Geological Survey, The Lyell Centre, Edinburgh, United Kingdom; 4 Department of Earth Science, University of Bergen, Bergen, Norway; 5 Department of Earth, Environmental and Planetary Sciences, Rice University, Houston, USA; 6 Institute for Geophysics, University of Texas at Austin, Austin, USA; 7 School of Earth and Environment, The University of Leeds, Leeds, United Kingdom; 8 Department of Animal Ecology and Systematics, Justus Liebig University, Giessen, Germany; 9 Institut de Physique du Globe de Paris, Sorbonne Paris Cité, Université Paris Diderot, Paris, France; 10 Departamento Geociencias Marinas y Ordenación del Territorio, Facultad de Ciencias del Mar, Universidad de Vigo, Vigo, Spain; 11 Université Grenoble Alpes, Université Savoie Mont Blanc, CNRS, IRD, IFSTTAR, and ISTerre, Le Bourget-du-Lac, France; 12 CRPG, UMR 7358, France. Also at: Université de Lorraine, ENSG, INP, Nancy, France; 13 Department of Geology, University of Patras, Patras, Greece; 14 Center for Tectonics, Resources, and Exploration (TRaX), Department of Earth Sciences, School of Physical Sciences, University of Adelaide, Adelaide, Australia; 15 Géosciences Montpellier, Université de Montpellier, Montpellier, France; 16 University of Hawai’i at Manoa, Hawai’i Institute of Geophysics and Planetology, Honolulu, USA; 17 University Centre for Earth and Space Sciences, University of Hyderabad, Hyderabad, India; 18 Universidade Federal de São Paulo, Departamento de Ciências do Mar, Sao Paulo, Brazil; 19 Department of Geology and Geoenvironment, National and Kapodistrian University of Athens, Athens, Greece; 20 School of Geography, Geology and the Environment, University of Leicester, Leicester, United Kingdom; 21 School of Energy Resources, China University of Geosciences, Beijing, China; 22 School of Geography Earth and Environmental Sciences, University of Birmingham, Birmingham, United Kingdom; 23 Department of Physical Sciences, Kingsborough Community College, City University of New York, New York, USA; 24 Laboratory of Geophysics and Seismology, Technological Educational Institute of Crete, Irakleio, Greece; 25 Graduate School “Human Development in Landscapes”, Christian-Albrechts-Universität zu Kiel, Kiel, Germany; 26 Department of Geology and Geophysics, Louisiana State University, Baton Rouge, USA; 27 Institute of Geology and Minerology, University of Cologne, Cologne, Germany; 28 Ifremer, Department of Marine Geosciences, Centre Bretagne, Plouzané, France; 29 Institute for Ecosystem Research, Christian-Albrechts-Universitat zu Kiel, Kiel, Germany; 30 Department of Earth and Atmospheric Sciences, Central Michigan University, Mount Pleasant, USA

Correction to: *Scientific Reports* 10.1038/s41598-019-40022-w, published online 28 February 2019

This Article contains errors in Reference 40 which is incorrectly given as:

Palyvos, N., Pantosti, D. & Zabci, C. Paleoseismological evidence of recent earthquakes on the 1967 Mudurnu Valley earthquake segment of the North Anatolian fault. *Bull. Seis. Soc. Am*. **97**, 1646–1661 (2007).

The correct Reference 40 appears below as Reference 1.
