# Supplementary material for: High-resolution record reveals climate-driven environmental and sedimentary changes in an active rift
Source: Sci Rep. 2019 Feb 28;9:3116. doi: 10.1038/s41598-019-40022-w (PMC6395724; doi:10.1038/s41598-019-40022-w)

High-resolution record reveals climate-driven environmental and sedimentary changes in an active rift

Lisa C. McNeill*, Donna J. Shillington, Gareth D. O. Carter, Jeremy D. Everest, Robert L. Gawthorpe, Clint Miller, Marcie P. Phillips, Richard E. Ll. Collier, Aleksandra Cvetkoska, Gino De Gelder, Paula Diz, Mai-Linh Doan, Mary Ford, Maria Geraga, Jack Gillespie, Romain Hemelsdaël, Emilio Herrero-Bervera, Mohammad Ismaiel, Liliane Janikian, Katerina Kouli, Erwan Le Ber, Shunli Li, Marco Maffione, Carol Mahoney, Malka L. Machlus, Giorgios Michas, Casey W. Nixon, Sabire Asli Oflaz, Abah P. Omale, Kostas Panagiotopoulos, Sofia Pechlivanidou, Simone Sauer, Joana Seguin, Spyros Sergiou, Natalia V. Zakharova, Sophie Green

*corresponding author: lcmn@noc.soton.ac.uk

Methods

Micropaleontology and Basin Environment

Microfossils (calcareous nannofossils, marine and non-marine diatoms, planktic and benthic foraminifer, dinoflagellate cysts, foraminifer test linings, freshwater algae coenobia and spores, and aquatic pollen and spores) were used to distinguish basin paleoenvironment, principally differentiating "marine" or "isolated". Table S1A and S1B give abundances of key microfossil groups: calcareous nannofossils and benthic foraminifer (marine indicators) and non-marine diatoms (isolated indicators) with depth in Hole M0079A which are plotted in Figure 2. Qualitative counts of calcareous nannofossils and non-marine diatoms are based on the Cascading Count Method of Styzen (1997). The numerical approximations associated with abundance (abundance: Barren (B), Very Rare (VR), Rare (R), Few (F), Common (C) and Abundant (A)) for calcareous nannofossils and non-marine diatoms are outlined below:

Calcareous Nannofossils: B = 0; VR = 1-5; R = 6-100; F = 101-1500; C = 1501-5000; A = 5001-10,000+

Non-marine Diatoms: B = 0; VR = 1; R = 2; F = 3-10; C = 11-20; A = 21-50+

The abundance of benthic foraminifer is represented by the number of individuals found in ~10 cc of wet sediment. Benthic foraminifer were counted in the >125 µm fraction. In the marine intervals, counts are generally higher than 100 individuals whereas in the isolated intervals the abundance of individuals in the >125 µm is generally lower than 10 individuals. In those intervals, the 63-125 µm was additionally screened for small foraminifer. Low total benthic foraminiferal abundances in the >63 µm fraction characterise the isolated interval.

Age Model

Age constraints were taken from shipboard biostratigraphic (calcareous nannofossils) and magnetostratigraphic analyses (Fig. 2; see also Shillington et al., in press).

Calcareous nannofossils provide three age markers (see main text and Figure 2 for details), based on analysis of samples from core catchers (approximately 3-m intervals) and additional in-core samples. Species identification, relative abundance and preservation were determined using a reflected light microscope at 1000x magnification under both plain and polarized light. The biozonation of Backman et al. (2012), developed for Miocene through Pleistocene calcareous nannofossils from low to middle latitudes, was applied to the calcareous nannofossils to determine geologic age. This biozonation was further calibrated with the most recent Geologic Time Scale of Ogg et al. (2016). The website www.mikrotax.org was consulted as necessary to aid in the identification of calcareous nannofossils. Due to the fluctuating environment within the basin, the syn-rift stratigraphy does not contain a continuous marine section, thus the LO of *P. lacunosa* and FO of *E. huxleyi* may not mark the true respective LO and FO. We note that *P. lacunosa* was identified in a coherent interval within a large-scale slump that defines Subunit 1-11 (Fig. 2; Shillington et al., in press) surrounded by intervals interpreted as isolated. The stratigraphic interval containing this marine calcareous

nannofossil assemblage is intact with no evidence for reworking. Therefore, this coherent interval of slumped sediments containing *P. lacunosa* is interpreted to represent a part of an older marine interval, most likely the time equivalent of the underlying marine Subunit 1-13.

Preliminary magnetostratigraphy analysis provides one age marker in Hole M0079A, the Brunhes-Matuyama chron boundary at 0.773 Ma at a depth of 665 mbsf (Fig. 2). A total of 532 discrete sediment cubic samples were collected from working halves at intervals of ~1.5 m throughout the borehole. All samples were demagnetised using alternating field (AF) treatment in 14 progressive field steps from 5 to 40 mT (with 5-mT increments) and from 40 to 100 mT (with 10-mT increments). Remanent magnetisation direction and intensity were measured before and after each demagnetization step using the horizontal pass-through super-conducting cryogenic rock magnetometer (SRM 755–4000, 2G Enterprises) at the University of Bremen. The inclination of the remanence after AF demagnetisation at 40 mT was used to determine the polarity of each sample (i.e., normal or reversed) and build a magnetostratigraphy downhole. The Gulf of Corinth is located at an average latitude of 38°8.45'N, and hence reversals of the Earth's magnetic field can be easily identified by distinct changes in inclination of the remanence vectors. Based on the geocentric axial dipole (GAD) assumption, the expected field inclination at the drill sites is 57.5°. Given the young age of the sediment and therefore the lack of major latitudinal motion of this site, we assume a constant expected inclination throughout the recovered sedimentary sequence. Magnetozones identified from the data were correlated to the Geomagnetic Polarity Time Scale – GPTS (Cande and Kent, 1995; Ogg et al., 2016) with the aid of biostratigraphic datums.

In addition to the above age markers, the age model was developed further by tying the Unit 1 subunit boundaries between the marine and isolated intervals to the eustatic sea level of Spratt and Lizecki (2016) (Fig. S1, Table S2). A sill depth of -60 m (the current depth of the Rion sill at the western end of the Gulf of Corinth; Perrisoratis et al., 2000) was used to determine the timing of the transitions between marine and isolated. The assumption that the sill depth remains constant is made for generating this age model – see below for further discussion on sill depths and their impact on sedimentation rates. All ages between these transitions were extrapolated linearly. Because the apparent LO of *P. lacunosa* was identified within a coherent block in a slumped interval (Subunit 1-11), and the source of this block interpreted as time correlative with

underlying Subunit 1-13, it is appropriate to correlate Subunits 1-10 to 1-12 to OIS 12 and the underlying marine interval (Subunit 1-13) to OIS 13. We note that in this case, the true position of LO of *P. Lacunosa* could be either above or below its identified position, and is marked as such on Figure 2.

Below a depth of ~545 mbsf (base Subunit 1-13 and interpreted OIS 13, estimated age 535 ka), marine intervals were thin or absent. Therefore, the age model developed from microfossil-based sea level correlation (and calculations of sedimentation rate, see below) could not be applied through to the base of the hole; however, the Brunhes-Matuyama chron boundary at 665 mbsf provides absolute age constraint near the base of the hole. Ages and depths in Hole M0079A based on this method are shown in Table S2.

Lithology and Facies Associations

The lithostratigraphy of the syn-rift sediments drilled during Expedition 381 were categorised into facies associations (FA; Shillington et al., in press). The FA were defined based on physical and biogenic features of the sediment, including bedding and lamination style, grain size, colour, body and trace fossils. A simplified version of the facies associations present in Hole M0079A (integrating related facies associations) is depicted in Figure 2 with accompanying key. The facies associations used in this paper are defined in Table S3.

Bioturbation Intensity

The degree of bioturbation applied to the cores in this study and represented on Figure 2 is a semi-quantitative assessment ranging from 0 (no bioturbation) to 6 (completely bioturbated), following the scheme of Taylor and Goldring (1993). Blank regions represent areas of no visible bioturbation.

Sedimentation Rate

In order to determine sedimentation rates, we used the ages determined above and decompacted sediment thicknesses. The rate calculated uses all sediment accumulated, i.e., hemipelagic and gravity flow deposits. Decompaction was based on porosities measured using the "moisture and density" technique on discrete 6 cm^3 samples spaced at ~1.5 m on cores from Hole M0079A. The wet and dry masses of these samples were measured before and after being dried in a convection oven at 60° ± 5°C for 24 hrs, respectively. The volume of dried sample was measured with a helium-displacement pycnometer. These measurements were then used to calculate the volume and mass of water originally in the samples, and the porosity of the samples (Table S4; Shillington et al., in press).

Next, we determined a smooth porosity function to use for decompaction based on these porosities. We first removed outliers by fitting a 2nd order polynomial to the measured porosities and discarding values with residuals greater than 1.5. We fit a 35th order polynomial to the remaining points. A high-order polynomial was required to capture the observed variations in porosity between marine and isolated subunits (Fig. S2, Table S5). Use of the higher order polynomial does result in mild fluctuations in the sediment accumulation rate within the marine and isolated intervals (Fig. 2). However, the order of the polynomial had little impact on the derived sedimentation rates.

The decompacted thickness of sediments from a given depth interval, T_i^*, was determined (Table S5) assuming that there is not alteration of the grains:

$$T_i^* = \frac{T_i(1-\phi_i)}{(1-\phi_i^*)}$$

where T_i and ϕ_i are the compacted thickness and porosity of a given interval, respectively, where the porosity is taken from the smooth function calculated above. ϕ_i^* is the initial porosity, and is assumed to be 56%, the porosity at the modern seafloor in Hole M0079A (Table S4; Shillington et al., in press). We tested sensitivity to changing the assumed initial porosity value and to using different initial porosities for the marine and isolated intervals (rather than the constant 56%). Case 1 involved initial porosities of 70% and 56% in the isolated and marine intervals, respectively. Case 2 involved initial porosities of 56% and 70% in the marine and isolated

intervals, respectively. In both cases, the same pattern of increased sedimentation rate during the isolated/glacial intervals is generated that we observe using the measured present-day initial 56% porosity throughout.

Sill depths and impact of changing sill depth on sedimentation rates

At the western mouth of the Gulf of Corinth, two shallow sills have been identified at similar depths: the Rion sill just east of Patras commonly used for basin level reconstructions (-60 m depth; Perissoratis et al., 2000) and the Acheloos-Cape Pappas sill bounding the Gulf of Patras farther west (-50-55 m depth plus 5-7 m Holocene sediment drape; Beckers et al., 2016; Piper and Panagos, 1980) (Fig. 1). These western sills are interpreted to have controlled flooding of the Gulf for at least the last 200 kyr (e.g., Beckers et al., 2016; Perissoratis et al., 2000), with good support for a sill at a depth of ~60 m provided by the ~12 ka age of the isolated-marine transition in shallow piston cores (e.g., Moretti et al., 2004). At the eastern end of the Gulf of Corinth, connection to the open ocean in the past may have been controlled by the Corinth Isthmus (Fig. 1), although the Isthmus is currently above sea level. Marine fauna in the vicinity of the Corinth Canal and Isthmus area have been identified and dated from ~400-200 ka (Collier and Thompson, 1991; Dia et al., 1997) with no more recent marine deposits identified. These are interpreted as indications the Isthmus has been above sea level since ~200 ka, but may have acted as a sill before ~200 ka. Prior to ~450 ka, the Isthmus may have been completely open to the Aegean, but with a sill farther east preventing full ocean connection. In terms of stability of the Rion sill depth, Perissoratis et al. (2000) suggest there are low subsidence rates in the area (Chronis et al., 1991) and a lack of fault offset of the terraces at the Rion sill, both of which support evidence for the sill depth changing little in the late Pleistocene.

The potential impact of varying sill depth on the age model and derived sedimentation rates has been investigated. As increasing sill depth (>60 m) will only serve to amplify the result of significantly higher sedimentation rates in isolated intervals relative to marine intervals (because the basin will experience lengthened marine intervals), we test only shallower sill depths. Depths of -30, -50 and -60 m are tested and plotted on Figure S3. We find that even for a sill as shallow

as -30 m, sedimentation rates are still consistently higher during isolated intervals relative to marine intervals.

References

Backman, J., Raffi, I., Rio, D., Fornaciari, E., Pälike, H. 2012. Biozonation and biochronology of Miocene through Pleistocene calcareous nannofossils from low and middle latitudes. Newsletters on Stratigraphy, 45 (3), 221-244.

Beckers, A., Beck., C., Hubert-Ferrari, A., Tripsanas, E., Crouzet, C., Sakellariou, D., Papatheodorou, G., and De Batist, M., 2016, Influence of bottom currents on the sedimentary processes at the western tip of the Gulf of Corinth, Greece. Marine Geol. 378, 312-332.

Cande, S.C., and Kent, D.V., 1995. Revised calibration of the geomagnetic polarity timescale for the Late Cretaceous and Cenozoic. J. Geophys. Res.: Solid Earth, 100(B4):6093–6095. doi:10.1029/94JB03098.

Chronis, G., Piper, D.J.W., Anagnostou, C., 1991. Late Quaternary evolution of the Gulf of Patras, Greece: tectonism, deltaic sedi- mentation and sea level change. Mar. Geol. 97, 191–209.

Collier, R. E. L. & Thompson, J., 1991. Transverse and linear dunes in an upper Pleistocene marine sequence, Corinth basin, Greece. Sedimentology 38, 1021-1040.

Dia, A.N., Cohen, A.S., O'Nions, R/K., and Jackson, J.A., 1997, Rates of uplift investigated through ^{230}Th dating in the Gulf of Corinth (Greece). Chemical Geology, 138, 171-184.

Moretti, I. et al. Sedimentation and subsidence rate in the Gulf of Corinth: what we learn from the Marion Dufresne's long-piston coring. Tectonics 336, 291-299 (2004).

Nixon, C.W., McNeill, L.C., Bull, J.M., Bell, R., Gawthorpe, R., Henstock, T., Christodoulou, D., Ford, M., Taylor, B.,Sakellariou, D., Ferentinos, G., Papatheodorou, G., Leeder, M., Collier, R., Goodliffe, A., Sachpazi, M., Kranis, H., 2016. Rapid spatiotemporal

variations in rift structure during development of the Corinth Rift, central Greece. Tectonics, 35(5), 1225–1248. https://doi.org/10.1002/2015TC004026

Ogg, J., Ogg, G., Gradstein, F., 2016. A Concise Geologic Time Scale 2016. Elsevier B.V., ISBN 978-0-444-63771-0.

Perissoratis, C., Piper, D. J. W., and Lykousis, V., 2000, Alternating marine and lacustrine sedimentation during late Quaternary in the Gulf of Corinth rift basin, central Greece. Marine Geol., 167, 391-411.

Piper, D.J.W., and Panagos, A.G., 1980, Surficial sediments of the Gulf of Patras. Thalassographica, 1(3), 5-20.

Shillington, D.J., McNeill, L.C., Carter, G., and Expedition 381 Scientists, in press, *Expedition 381 Preliminary Report: Corinth Active Rift Development*. International Ocean Discovery Program.

Spratt, R.M., and Lisecki, L.E., 2016. A Late Pleistocene sea level stack. Climate of the Past, 12, 1079-1092.

Styzen, M.J., 1997. Cascading Counts of Nannofossil Abundance. Journal of Nannoplankton Research, Vol. 19, No. 1.

Taylor, A.M. and Goldring, R., 1993. Description and analysis of bioturbation and ichnofabric. Journal of the Geological Society of London, 150:141-148. doi: https://doi.org/10.1144/gsjgs.150.1.0141

Taylor, B., Weiss, J.R., Goodliffe, A.M., Sachpazi, M., Laigle, M., and Hirn, A. 2011. The structures, stratigraphy and evolution of the Gulf of Corinth rift, Greece. Geophysical Journal International, doi: 10.1111/j.1365-246X.2011.05014.x

Supplementary Tables and Figures

Supplementary Table S1A. Qualitative counts of calcareous nannofossils and and non-marine diatoms; S1B. Counts of benthic foraminifer. Both with depth in Hole M0079A. (See separate excel file).

Supplementary Table S2. Depths in Hole M0079A and ages of subunit boundaries from correlation with the eustatic sea level curve of Spratt and Lisecki (2016). Basin paleoenvironment and Oxygen Isotope Stage interpretation also shown.

Supplementary Table S3. Lithological facies association (FA) descriptions used in Figure 2.

Supplementary Table S4. Original porosity data with depth from Hole M0079A core samples. (See separate excel file).

Supplementary Table S5. Age from age model, fitted porosity data, compaction-corrected thicknesses and depths and calculated decompaction-compensated sedimentation rates with depth in Hole M0079A. (See separate excel file).

Supplementary Figure S1. Sea level curve (Spratt and Lisecki, 2016) with Rion sill depth (60 m below sea level) used to generate age model.

Supplementary Figure S2. Porosity data from “moisture and density” measurements with polynomial fit to data used for Hole M0079A, resulting decompacted depths, and sedimentation rates with depth. Grey bars mark marine intervals/subunits.

Supplementary Figure S3. Plot showing impact of changing basin sill depth on decompaction-corrected sedimentation rates.

Supplementary Figure S4. *Ewing* Line 41 (Taylor et al., 2011) across Site M0079 with interpreted horizons from Nixon et al. (2016).

Table S2

Depth (mbsf)	Subunit	Paleoenvironment interpretation	OIS	Age (ka)
0	1	Marine	1	0
31.94	1	Marine	1	
31.94	1	Isolated		12.59
149.1	2	Isolated		
149.1	2	Marine	5	70.39
183.88	3	Marine	5	
183.88	3	Isolated	5	129.34
265.76	4	Isolated		
265.76	4	Marine	7	187.80
307.18	5	Marine	7	
307.18	5	Isolated		245.30
383	6	Isolated		
383	6	Marine	9	276.36
406.02	7	Marine	9	
406.02	7	Isolated		335.10
449.02	8	Isolated		
449.02	8	Marine	11	365.64
462.69	9	Marine	11	
462.69	9	Isolated		420.35
484.48	10	Isolated		
484.48	10	Slumped unit		
506.4	11	Slumped unit		
506.4	11	Isolated		
524.98	12	Isolated		
524.98	12	Marine	13	467.27
543.8	13	Marine	13	
543.8	13	Isolated		534.61

Table S3

Facies Association	Definition
1	Homogeneous mud
2	Greenish, grey mud with dark grey to black mud to sand beds and laminations
3	Light grey to white laminations alternating with mud and silt beds
4	Laminated greenish grey to grey mud with mud beds
5	Greenish grey mud with homogeneous cm-thick grey mud beds
6	Green bedded partly bioturbated mud, silt and sand
10	Interbedded mud/silt and dm-thick sand beds
11	Interbedded mud/silt and cm-thick sand beds
12	Light grey to buff homogenous to weakly stratified, bioturbated mud
13	Contorted bedding and mud-supported sands and conglomerates

Figure S1

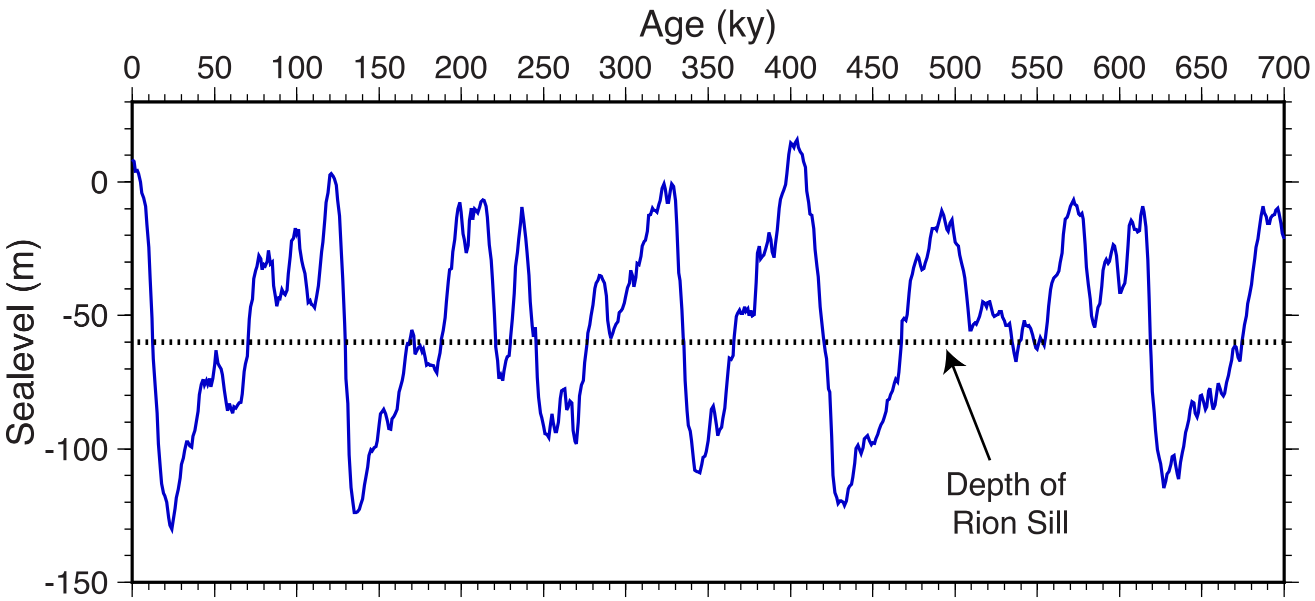
Age (ky)
0
50
100
150
200
250
300
350
400
450
500
550
600
650
700
Sealevel (m)
0
-50
-100
-150
Depth of
Rion Sill

McNeill et al, Figure S2

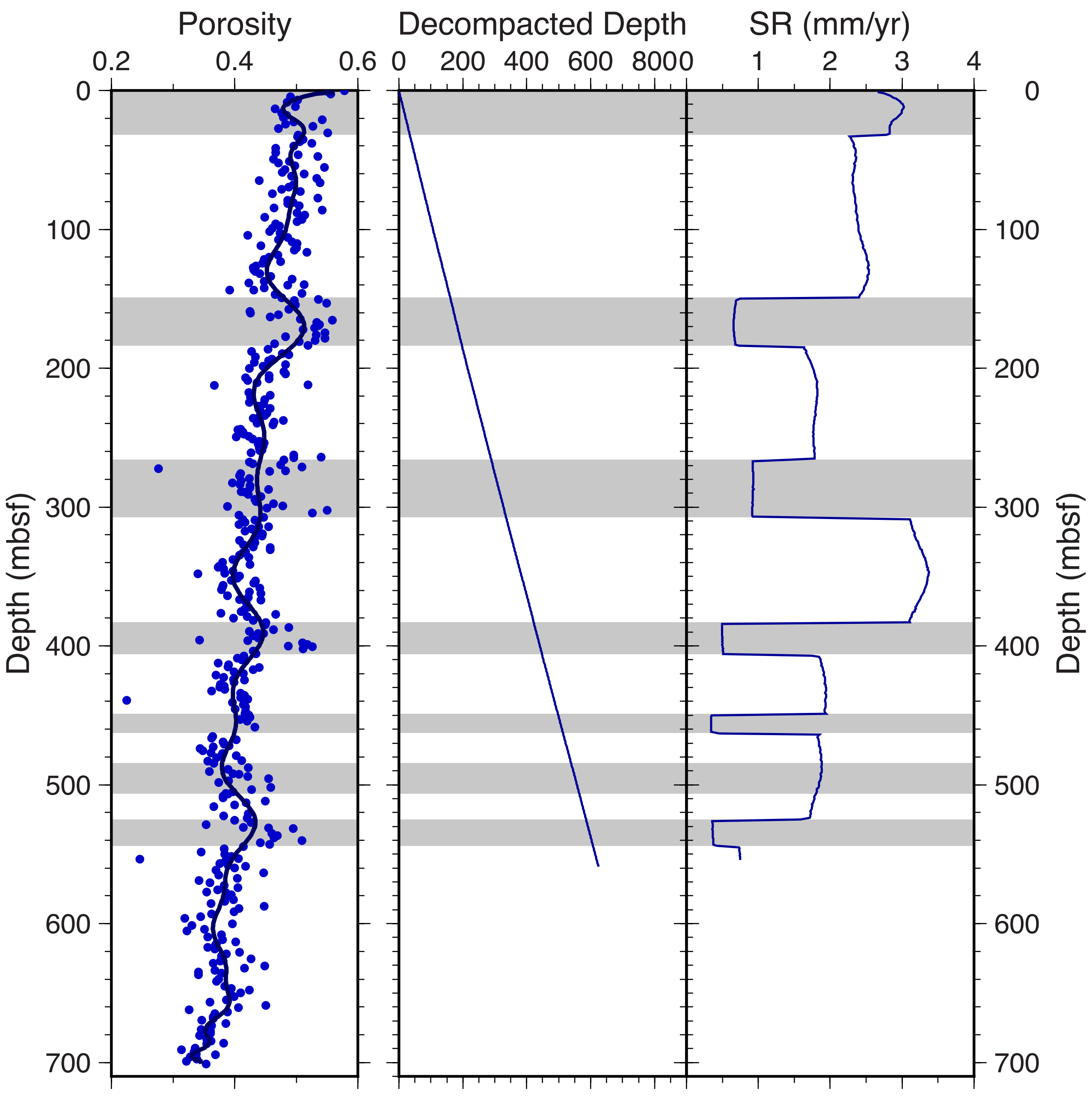

McNeill et al., Figure S3

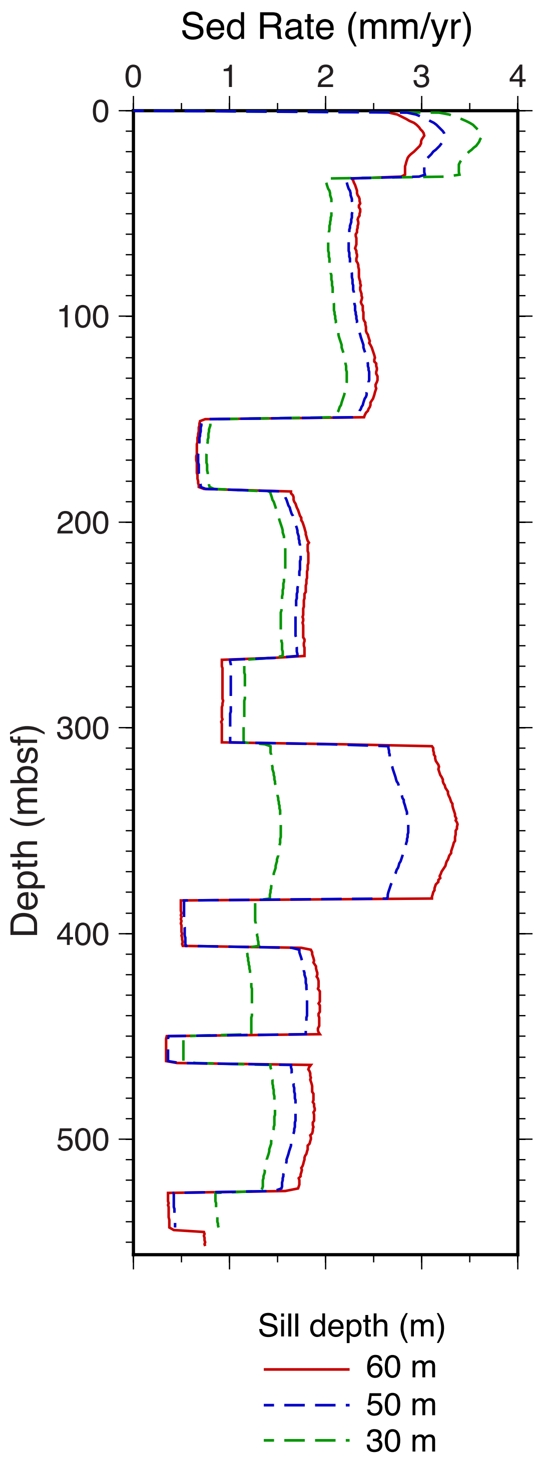

McNeill et al., Figure S4

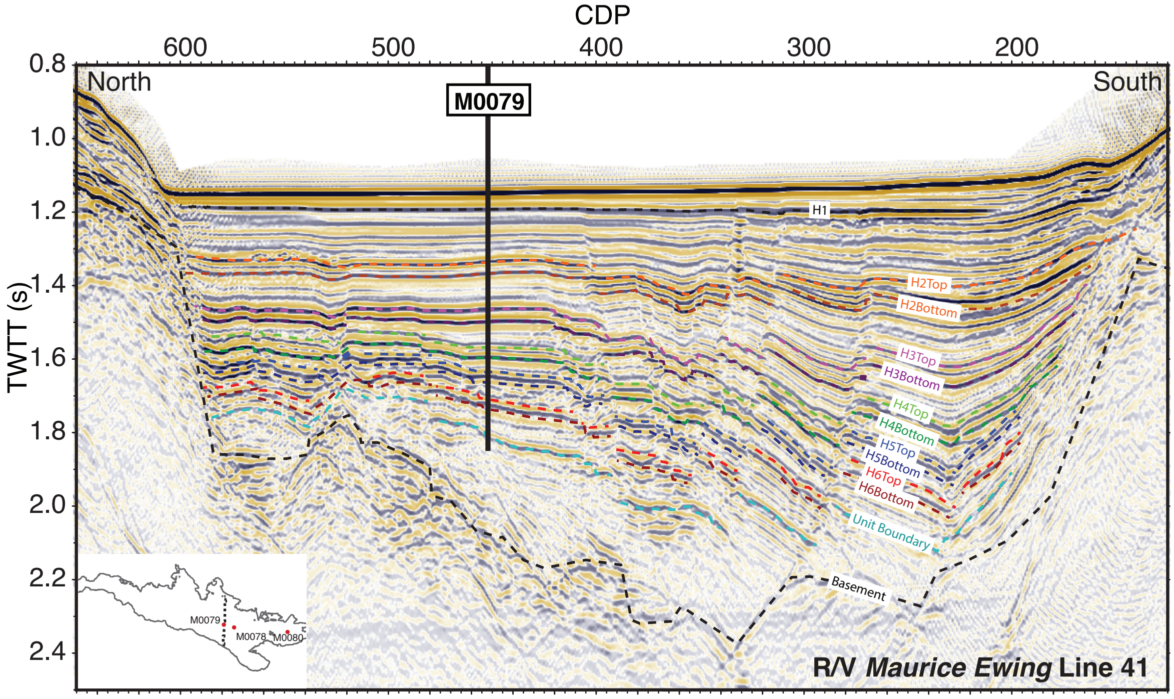

Supplement: Supplementary file 1 — Supplementary information [file 41598_2019_40022_MOESM1_ESM.pdf]
